# A triple-sugar regulated *Salmonella* vaccine protects against *Clostridium perfringens*-induced necrotic enteritis in broiler chickens

**DOI:** 10.1016/j.psj.2021.101592

**Published:** 2021-11-17

**Authors:** Shifeng Wang, Charles L. Hofacre, Soo-Young Wanda, Jingyu Zhou, Richard A. Callum, Bob Nordgren, Roy Curtiss

**Affiliations:** ⁎ Department of Infectious Diseases and Immunology, College of Veterinary Medicine, University of Florida, Gainesville, FL 32611, USA; † College of Veterinary Medicine, University of Georgia, Athens, GA 30602, USA; ‡ Southern Poultry Research Group, Inc., Watkinsville, GA 30677, USA; § Curtiss Healthcare Inc, Alachua, FL 32615, USA

**Keywords:** *Clostridium perfringens*, necrotic enteritis, *Salmonella*, vaccine, NetB, PlcC

## Abstract

Gram-positive *Clostridium perfringens* type G, the causative agent of necrotic enteritis (**NE**), has gained more attention in the poultry industry due to governmental restrictions on the use of growth-promoting antibiotics in poultry feed. Our previous work has proved that regulated delayed lysis *Salmonella* vaccines delivering a plasmid encoding an operon fusion of the nontoxic C-terminal adhesive part of alpha toxin and a GST-NetB toxin fusion were able to elicit significant protective immunity in broilers against *C. perfringens* challenge. We recently improved our *S.* Typhimurium antigen delivery vaccine strain by integrating a rhamnose-regulated O-antigen synthesis gene enabling a triple-sugar regulation system to control virulence, antigen-synthesis and lysis in vivo traits. The strain also includes a Δ*sifA* mutation that was previously shown to increase the immunogenicity of and level of protective immunity induced by *Salmonella* vectored influenza and *Eimeria* antigens. The new antigen-delivery vaccine vector system confers on the vaccine strain a safe profile and improved protection against *C. perfringens* challenge. The strain with the triple-sugar regulation system delivering a regulated lysis plasmid pG8R220 encoding the PlcC and GST-NetB antigens protected chickens at a similar level observed in antibiotic-treated chickens. Feed conversion and growth performance were also similar to antibiotic-treated chickens. These studies made use of a severe *C. perfringens* challenge with lesion formation and mortality enhanced by pre-exposure to *Eimeria maxima* oocysts. The vaccine achieved effectiveness through three different immunization routes, oral, spray and in drinking water. The vaccine has a potential for application in commercial hatcher and broiler-rearing conditions.

## INTRODUCTION

*Clostridium perfringens* is an enteric pathogen affecting humans and food animals including chickens, pigs, cattle, and horses. It can be categorized into 7 types according to the toxins produced (Rood et al., 2018). Type G strains are the primary etiologic agent to cause necrotic enteritis (**NE**) in poultry, mainly in the broiler industry. Acute clinical infections with *C. perfringens* that are often intensified by co-infection with *Eimeria* sp. can lead to high mortality rates up to 50% in flocks sometimes (Timbermont et al., 2011). Subclinical infections can impair growth rates, decrease feed conversion rates, reduce weight gain, and lead to significant losses. The subclinical form of NE is a worldwide problem with an average of 80% of the flocks having had *Clostridium* diagnosed (Verleyen, 2010). There are increased productivity losses of NE in all regions of the world. NE costs the international poultry industry 6 billion US dollars per year in production losses and costs for control measures (Wade and Keyburn, 2015).

Besides good management, the inclusion of antibiotics in feed is the most commonly used method to promote growth and prevent this disease by improving the intestinal health of poultry. However, the widespread use of antibiotics leads to the spread of antibiotic-resistance genes in bacteria in the environment. *C. perfringens* strains have been found to be resistant to medically important antibiotics for humans: tetracycline, virginiamycin, penicillin, clindamycin, vancomycin, ceftriaxone, and erythromycin (Slavic et al., 2011; Wei et al., 2020). Besides, *C. perfringens* has been found to be resistant to bacitracin (Slavic et al., 2011), a narrow-spectrum antibiotic used to prevent and control NE, increase rate of weight gain and improve feed efficiency in the poultry industry (Prescott et al., 1978). This resistance poses a challenge for the efficacy of bacitracin in the future. Due to the concern of increased antibiotic-resistant bacteria, use of growth-promoting subtherapeutic antibiotics in animal feeds has been phased out and banned in Europe since 2006. However, an increased incidence of NE has been associated with the withdrawal of antibiotic growth promoters from poultry feed (Timbermont et al., 2011). Infections of poultry with *C. perfringens* have likewise increased markedly (Van Immerseel et al., 2004). Thus, the control of this pathogen has gained more attention. Several options, including the use of acidifiers, phytobiotics, probiotics, minerals, fatty acids, and plant extracts have been tried to prevent *C. perfringens* infection in poultry (Van Immerseel et al., 2004; Caly et al., 2015). Among them, probiotics have been used to prevent subclinical NE (Khalique et al., 2020) but usually need to be administrated throughout all chicken growth stages. Besides, the beneficial effects of probiotics vary under farm conditions (Adhikari et al., 2020; Khalique et al., 2020). Therefore, it is necessary to find other cost-effective methods in order to prevent this disease, reduce economic losses and the spreading of antibiotic resistance genes. A vaccine against *C. perfringens* to prevent NE will be one of the best options.

Many antigens, such as TpeL, perfringolysin O, hypothetical protein, pyruvate:ferredoxin oxidoreductase, elongation factor Tu, glyceraldehyde-3-phosphate dehydrogenase, endo-beta-N-acetylglucosaminidase, mucinase, metallopeptidase, phosphoglyceromutase, pilin structural subunits, fructose-1,6-biphosphate aldolase and lipoteichoic acid, have been identified as potential protective antigens against *C. perfringens* (Mot et al., 2014; Duff et al., 2019; Lepp et al., 2019; Katalani et al., 2020; Wenzel et al., 2020). Two nontoxic antigens, PlcC (carboxy-terminal fragment of α-toxin) and GST-NetB (fusion of Glutathione-S-Transferase [**GST**] with NetB toxin) have been used in our lab to develop a vaccine against *C. perfringens*. The GST tag promotes increased protein yields, solubility and stability of the protein, and avoids intracellular digestion. GST also provokes the strongest antibody response to the carried antigen compared to other carriers (Yip et al., 2001). Antibodies against α-toxin can inhibit *C. perfringens* growth (Zekarias et al., 2008), affect the membrane-binding of toxin (Stevens et al., 2004) and provide partial protection against *C. perfringens* challenge (Stevens et al., 2004; Zekarias et al., 2008). NetB is an essential pore-forming toxin for NE in chickens (Keyburn et al., 2008). Vaccination with NetB as toxoid, detoxified subunit, or chimeric protein, could provide protection from the development of NE in chickens (Mot et al., 2014; Rood et al., 2016). It has been delivered by *Salmonella*, plant, non-virulent *C. perfringens* strain or in nanoparticles (Jiang et al., 2015; Lillehoj et al., 2017; Mishra and Smyth, 2017; Hunter et al., 2019; Wilde et al., 2019; Hoseini et al., 2021; Mauri et al., 2021). We previously showed that a regulated delayed lysis strain χ11802 delivering a plasmid pYA5112 encoding an operon fusion of PlcC and GST-NetB could induce partial protection against *C. perfringens* challenge immunized with a 2-dose regime in which chickens were immunized at 3- and 17-d of age (Jiang et al., 2015; Wilde et al., 2019). Multiple dosage vaccination regimens are not preferred for use in the broiler (Mot et al., 2014). A single vaccination regimen for day-of-hatch chicks should enable chicken to develop immunity to confer sufficient protection in the critical window of time when NE is most likely to occur. Thus, it was desirable to improve the candidate vaccine χ11802(pYA5112). Several strategies to enhance vaccine effectiveness are to improve the vaccine vector, include additional protective antigens or use alternative immunization regimes. This work focuses on the improvement of the *Salmonella* vaccine vector strain. Two-sugars, arabinose and mannose, are used to regulate the virulence traits of strain χ11802. In this study, we describe the development of a triple-sugar regulated vaccine vector system with improved performance attributes compared to the double-sugar regulated vaccine vector system. The new improved vaccine vector system delivering PlcC and GST-NetB antigens induced protection against severe challenges augmented by prior *Eimeria* infection by different *C. perfringens* strains.

## MATERIALS AND METHODS

### Bacterial Strains, Plasmids, Media, and Growth Conditions

Bacterial strains and plasmids used in this study are listed in Table 1. Plasmid pYA3681 is a lysis vector encoding arabinose-regulated *murA* and *asdA* expression and C2-regulated synthesis of antisense *asdA* and *murA* mRNA transcribed from the P22 P_R_ promoter (Kong et al., 2008). It has the P_trc_ promoter to direct antigen-encoding gene transcription. Plasmid pG8R220 is derived from pYA3681 with an operon fusion of *plcC* and *gst-netB* under the control of the P_trc_ promoter. It is similar to the previously reported plasmid pYA5112 (Jiang et al., 2015), with a *Hind*III site replacing a *Pst*I site. All *S.* Typhimurium vaccine strains were grown at 37°C in Luria-Bertani broth (**LB**) broth or on LB agar with necessary supplements (Wang et al., 2010). Based on accumulated results demonstrating complete biological containment and safety of our recombinant self-destructing protective immunity enhanced attenuated *Salmonella* vaccine vectors encoding for delivery of protective antigens from various bacterial, viral and parasite pathogens in newborn, pregnant, malnourished, and immune deficient SCID mice, in multiple studies with mice, chickens, pigs and in a human phase 1 trial with no adverse events, bacteremia or shedding of viable recombinant vaccine cells in stools over a 12-d period at oral doses of 10^10^ CFU, the NIH Office of Science Policy and Recombinant Advisory Committee granted permission in 2016 for us to evaluate our genetically modified vaccines at Biosafety level 1 containment and under settings simulating commercial rearing for farm animals and in out-patients for human trials. The UF Biosafety Committee confirmed this permission based on presence of certain mutations in vaccine vector strains conferring these safety and beneficial attributes. The Southern Poultry Research Group, Inc (**SPRG**) IBC also approved this level of containment for the studies undertaken in their facilities. Carbohydrate-free purple broth medium (BD Biosciences, Franklin Lakes, NJ) was used to evaluate the effects of sugars on growth, gene expression, and LPS profile. When required, media were supplement with chloramphenicol (Cm, 30 µg/mL), 2-6-diaminopimelic acid (DAP, 50 µg/mL) (Nakayama et al., 1988)^,^ L-arabinose (Ara, 0.1% v/v), L-rhamnose (Rha, 0.1% v/v), and mannose (Man, 0.1% v/v). All chemicals were from Sigma-Aldrich (St. Louis, MO). Bacterial growth curves were obtained using optical density measurements with a GENESYS 10 UV Spectrophotometer (Thermo Scientific) and by plating serial dilutions of bacterial cultures on LB agar with supplements. LB agar without NaCl and containing 5% sucrose was used for *sacB* gene-based counter selection in allelic exchange to generate mutations (Wang et al., 2010). *C. perfringens* strains were grown anaerobically in thioglycollate broth supplemented with 5 % beef extract at 37°C for 15 h.

**Table 1:** Strains and plasmids used in this research.

Strain or plasmid	Relevant characteristics/genotype	Source or reference
Strains		
*S. enterica* serovar Typhimurium		
χ3761	Wild-type UK-1	(Wang et al., 2010)
χ11802	ΔP_murA25_::TT *araC* P_araBAD_*murA* Δ*asdA27*::TT *araC* P_araBAD_*c2* Δ(*wza-wcaM*)-*8* Δ*pmi-2426* Δ*relA198*::*araC* P_araBAD_*lacI* TT Δ*recF126*	(Jiang et al., 2015)
χ12341	ΔP_murA25_::TT *araC* P_araBAD_*murA* Δ*asdA27*::TT *araC* P_araBAD_ *c2* Δ(*wza-wcaM*)-*8* Δ*pmi-2426* Δ*relA197*::*araC* P_araBAD_*lacI* TT Δ*recF126* Δ*sifA26* Δ*waaL46* Δ*pagL64*::TT *rhaRS* P_rhaBAD_*waaL*	This study
*Clostridium perfringens* Type G		
CP4	Wild type, NetB+ TpeL+	(Thompson et al., 2006)
CP6	Wild type, NetB+ TpeL+	(Hofacre et al., 2018)
Plasmids		
pRE112	*sacB mobRP4* R6K *ori*; Cm^+^	(Wang et al., 2010)
pYA3716	Suicide vector to generate Δ*sifA26* mutation in pRE112	(Ashraf et al., 2011)
pYA3879	Suicide vector to generate Δ*relA197*::*araC* P_araBAD_ *lacI* TT mutation in pRE112	(Wang et al., 2010)
pYA4900	Suicide vector to generate Δ*waaL46* mutation in pRE112	(Kong et al., 2011b)
pYA5377	Suicide vector to generate Δ*pagL64*::TT *rhaRS* P_rhaBAD_*waaL* mutation in pRE112	This study
pYA3681	Lysis vector, pBR *ori, araC* P_araBAD_ SD-GTG *murA*, SD-GTG *asd*, P22 P_R_ antisense RNA, P_trc_ promoter	(Kong et al., 2008)
pYA5112	Lysis vector, Asd^+^, *plcC*, and *gst-netB* fragment operon fusion in pYA3681, *bla*-SSopt	(Jiang et al., 2015)
pG8R220	Lysis, Asd^+^, *plcC*, and *gst-netB* fragment operon fusion in pYA3681, *bla*-SSopt, similar to previous reported pYA5112, with a HindIII site replacing a PstI site.	This study

### Strain Construction and Characterization

The protective immunity enhanced *Salmonella* vaccine (**PIESV**) strains χ11802 (Jiang et al., 2015) and strain χ12341, double- and triple-sugar regulated strains, were designed to allow regulated delayed lysis and attenuation in vivo. Briefly, suicide vectors containing the nucleotide sequences designed to introduce defined deletion or defined deletion-insertion mutations were used to construct strain χ12341 (Wang et al., 2010). The suicide vectors, pYA3879 (Wang et al., 2010), pYA3716 (Ashraf et al., 2011), pYA4900 (Kong et al., 2011b) and pYA5377 were used to introduce mutations Δ*relA197*::*araC* P_araBAD_ *lacI* TT, Δ*sifA26,* Δ*waaL46,* and Δ*pagL64*::TT *rhaRS* P_rhaBAD_ *waaL,* respectively*.* The presence of mutations Δ*relA197*::*araC* P_araBAD_ *lacI* TT*,* Δ*sifA26,* Δ*waaL46,* and Δ*pagL64*::TT *rhaRS* P_rhaBAD_ *waaL,* were verified by PCR using corresponding primers described elsewhere (Wang et al., 2010; Ashraf et al., 2011; Kong et al., 2011b). The presence of the Δ*asdA27*∷TT *araC* P_araBAD_ *c2* mutation in *Salmonella* was confirmed by its dependence on DAP for growth (Nakayama et al., 1988). The presence of the ΔP_murA25_∷TT *araC* P_araBAD_ *murA* mutation was verified by its dependence on arabinose for growth (Kong et al., 2008). The presence of Δ*pagL64*::TT *rhaRS* P_rhaBAD_ *waaL* and Δ*pmi-2426* were examined by using silver-stained LPS profile as previously described (Wang et al., 2010). Other phenotype characterizations associated with mutations in the strains were described elsewhere (Jiang et al., 2015). After transferring control plasmid pYA3681 and antigen expression plasmid pG8R220 into vector strains χ11802 and χ12341, all the genotypes were verified using corresponding primers. All *Salmonella* vaccines employed the balanced-lethal vector-host concept we developed for stable plasmid maintenance (Nakayama et al., 1988) to ensure that live PIESVs will be sensitive to all antibiotics and thus unable to disseminate antibiotic resistance. Plasmid stability was determined in LB medium under nonselective condition (presence of arabinose and DAP) for 50 generations (Juárez-Rodríguez et al., 2012).

### Sodium Dodecyl Sulfate-Polyacrylamide Gel Electrophoresis and Immunoblots

To evaluate protein synthesis, vaccine strains were grown with aeration at 37°C to an optical density at 600 nm (OD_600_) of 0.8 with continued growth for 4 h after adding 1 mM isopropyl β-d-1-thiogalactopyranoside (**IPTG**). Equal numbers of cells were collected and subjected to sodium dodecyl sulfate-polyacrylamide gel electrophoresis (**SDS-PAGE**) gels for separation of proteins by electrophoresis (Wang et al., 2010). Proteins were transferred onto nitrocellulose membranes. The blots were evaluated for the syntheses of specific proteins using indicated anti-sera as previously described (Wang et al., 2010). Anti-LacI (Wang et al., 2010), anti-PlcC (Zekarias et al., 2008), anti-NetB (Jiang et al., 2015) sera were generated by immunization of rabbits using corresponding proteins and stocked at −20°C. Anti-GroEL antibody (G6532) was purchased from Sigma-Aldrich.

### Chicken Husbandry, Groups, and Treatment

All animal work at the SPRG was conducted in conformance with their Animal Use Guidelines that were approved by their Institutional Animal Care and Use Committee (**IACUC**) (Title, Comparative Efficacy of Curtiss Healthcare Necrotic Enteritis Vaccine Administered by Gavage at One Day of Age for the Control of Necrotic Enteritis caused by *Clostridium perfringens* in Broiler Chickens). Since infection and severe disease due to NE is only routinely observed as a consequence of co-challenge with *Eimeria* oocytes and *C. perfringens* that can cause mortality in un- and undervaccinated control groups, the approved protocol recognized that mortality in some birds used would be likely. The chicken experiments were carried out in the SPRG IACUC-approved facility (Building 2, 96 Roquemore Rd. Athens, GA 30607). The building temperature's range was maintained at an appropriate temperature for the age of the birds as per the Cobb primary breeder guidelines (Cobb-Vantress, Cleveland, GA). Feed and water were given ad libitum.

#### Experimental Ration

An unmedicated chicken starter compounded with feedstuffs commonly used in the United States was formulated. The diet was representative of a local commercial formulation and calculated analyses met or exceeded NRC broiler starter requirements. Experimental treatment feeds were prepared from this basal starter feed. Quantities of all basal feed used to prepare treatment batches of feed were documented. Treatment feeds were mixed at SPRG to assure a uniform distribution of the respective test article. The feed was transferred to Building 2 and distributed among cages of the same treatment. The resulting ration (in mash form) was fed during the study.

#### Animals

Day-of-hatch male Cobb 500 broiler chicks were obtained from Cobb-Vantress, Cleveland, GA. At the hatchery, the birds were sexed and had received routine vaccinations for Marek's Disease by in ovo inoculation (Merial/Boehringer Ingelheim Animal Health, Gainesville, GA). The healthy appearing chicks were used in the study. Papers or swabs from bottom of all chick boxes were cultured for presence of *Salmonella* on *Salmonella* selective agar medium.

#### Housing

Upon arrival, 8 chicks were placed per cage in Petersime battery cages in Building 2. At placement, all birds were fed with the starter feed. Building 2 is an insulated, concrete-floored, metal structure that measures 40 ft by 100 ft in a north-south direction. The floor space per animal was 0.63 square feet/bird. The feeder/water space per bird was 8 birds/24 × 3.5 inch feeder/water trough. A thermostatically controlled gas furnace/air conditioner maintained uniform room temperature. Even light illumination was provided.

### Procedures

#### Bird Allocation and Cage Randomization

The study began when the birds were placed (day-of-hatch) (DOT 0) at which time they were allocated to experimental cages randomized with regard to treatment. No birds were replaced during the study. Each treatment had 6 cages (48 birds total per treatment group to ensure statistical significance of results observed). Each trial was limited to only being able to compare 7 groups of birds such that the facilities did not permit all the comparison groups that might have been useful to include for each Trial.

#### Vaccine Preparation and Administration

For animal experiments, vaccine strains, 1:50 diluted into LB broth with necessary supplements from culture grown statically with the same media overnight at 37°C, were grown with aeration on a shaker at 180 rpm at 37°C to an OD_600_ of 0.8 to 0.9. Bacterial cells were harvested at room temperature by centrifugation and resuspended in buffered saline with 0.01% gelatin (**BSG**) (Ashraf et al., 2011). For vaccine treatment for the oral gavage method, at DOT 0 each chick was orally gavaged with 0.1 mL of the different vaccines ∼5 × 10^8^ CFU/chick unless otherwise specified. For spray method at DOT 0, a box of 48 chicks was coarse sprayed with a hand-held sprayer with a dose of 0.25 mL/chick of the vaccine at ∼5 × 10^8^ CFU/chick. The same amount of vaccine was given in non-chlorinated drinking water during the first days of life. All vaccines suspended in BSG were returned to the SPRG Laboratory on ice for re-titration and validation of purity after administration***.***

#### Trial 1

This trial was to compare the protection levels conferred by a double-sugar regulated strain χ11802 and a triple-sugar regulated strain χ12341 delivering the same plasmid pG8R220. The study consisted of 48 cages starting with 384 chicks. Each treatment was replicated in 6 blocks of 8 chicks per cage. There were 6 treatment groups. Treatment 1 was a nonmedicated, no challenge group (NV/NCh group). Treatment 2 was a nonmedicated, *C. perfringens* challenge group (NV group). Treatment 3 was given ∼5 × 10^8^ χ11802(pG8R220). Treatment 4 was given ∼5 × 10^8^ χ12341(pG8R220). Treatment 5 was given ∼5 × 10^8^ χ12341(pYA3681) (empty Vector control). Treatment 6 was given Bacitracin Methylene Disalicylate (**BMD**, Zoetis, NJ) 50 g/t (BMD). Chickens in treatment groups 2–6 were prechallenged with *E. maxima* oocytes and later challenged with *C. perfringens* strain CP6 (see below)*.*

#### Trial 2

This trial was to compare the protections conferred by χ12341(pG8R220) with different doses. The study consisted of 56 cages starting with 448 chickens total. There were 7 treatment groups. Treatment 1 was the NV/NCh group. Treatment 2 was the NV group. Treatment 3 was given ∼5 × 10^8^ χ12341(pG8R220) (oral high dose, HD Gavage). Treatment 4 was given ∼1 × 10^8^ χ12341(pG8R220) (oral intermediate dose, MD Gavage). Treatment 5 was given ∼5 × 10^7^ χ12341(pG8R220) (oral low dose, LD Gavage). Treatment 6 was given ∼5 × 10^8^ empty vector. Treatment 7 was the BMD group*.* Chickens in treatment groups 2–7 were prechallenged with *E. maxima* oocytes and later challenged with *C. perfringens* strain CP6 (see below)*.*

#### Trial 3

This trial was to compare the protections conferred by χ12341(pG8R220) with different immunization routes and doses. The study consisted of 56 cages starting with 448 chickens total. There were 7 treatment groups. Treatment 1 was the NV/NCh group. Treatment 2 was the NV group. Treatment 3 was orally gavaged with ∼5 × 10^8^ χ12341(pG8R220) (HD Gavage). Treatment 4 was gavaged with ∼1 × 10^8^ χ12341(pG8R220) (MD Gavage). Treatment 5 was sprayed ∼5 × 10^8^ χ12341(pG8R220) (HD Spray). Treatment 6 was given ∼5 × 10^8^ χ12341(pG8R220) in non-chlorinated drinking water (HD Drinking Water). Treatment 7 was BMD group***.*** Chicken in treatments 2–7 were prechallenged with *E. maxima* oocytes and later challenged with *C. perfringens* strain CP4 (see below)*.*

#### Weights of Chickens

All birds were weighed on DOTs 0, 14, 21, and 28. Feed was weighed on DOT 0 and the remaining feed was weighed on DOTs 14, 21, and 28.

#### Disease Induction

On DOT 14, all birds except those in the NV/NCh group were orally inoculated with ∼5,000 oocysts of *Eimeria maxima* since *E. maxima* oocysts cause more severe NE including higher mortality than *E. acervulina* (Hofacre et al., 1998, 2018). Starting on DOT 19 all birds (except Treatment 1) was orally given a broth culture of *C. perfringens* ∼10^8^ CFU/mL. There was no feed removed in this study prior to oral inoculations. The birds were administered 0.1 mL by oral gavage of a fresh broth culture once daily for 3 d (on DOTs 19, 20, and 21). Pens were checked daily for mortality. Moribund birds were euthanized and calculated as mortality.

#### *C. perfringens* Challenge Growth

The challenge strains used were *C. perfringens #6* (CP6) and *C. perfringens #4* (CP4), both of them previously shown to cause NE in broiler chicks (Thompson et al., 2006; Hofacre et al., 2018). Strains were inoculated into 1 liter of thioglycollate broth supplemented with 5% beef extract and incubated at 37°C for 15 h. Fresh broth cultures were prepared and used daily.

#### NE Intestinal Lesion Scoring

On DOT 21, three birds from each cage four hours post third *C. perfringens* challenge were selected, euthanized, weighed, and examined for the presence and degree of NE lesions. The scoring was based on a 0 to 3 score, with 0 = normal, 1 = mild (slight mucus covering and loss of tone, thin wall or friable), 2 = moderate (focal necrosis or ulceration), and 3 = marked (severe, sloughed mucosa with presence of blood in the lumen) (Hofacre et al., 1998; Richardson et al., 2017). Mean lesion scores were based on lesion assessments in surviving birds. Mortality was calculated separately. Dead birds were necropsied by a veterinarian experienced in NE challenge studies so that only dead birds that display NE are counted as the result of the *C. perfringens* challenge.

#### Salmonella

On the day chicks were received from the hatchery, swabs from the bottoms of all chick boxes were cultured for the presence of *Salmonella*. On DOT 21 each cage's dropping pan was swabbed for *Salmonella* at the termination of study. One swab was used for all pans in a treatment. All the swabs were confirmed negative for *Salmonella*. This indicated the absence of *Salmonella* in chicks as received from the hatchery and the absence of viable vaccine cells at the end of the study.

#### Statistics

Means for cage weight gain, feed consumption, feed conversion (adjusted for mortality: feed consumed/[final live weight + mortality weight]), NE lesion scores, and NE mortality were calculated. Statistical evaluation of the data was performed using a STATISTIX (Analytical Software, Tallassee, FL). Data were analyzed using one-way nonparametric analysis of variance (**ANOVA**) to compare the means with the follow-up Tukey multiple comparison test at a significant level of 0.05.

## RESULTS

### Development of a Triple-Sugar Regulated Vaccine Vector System

**Figure 1:**
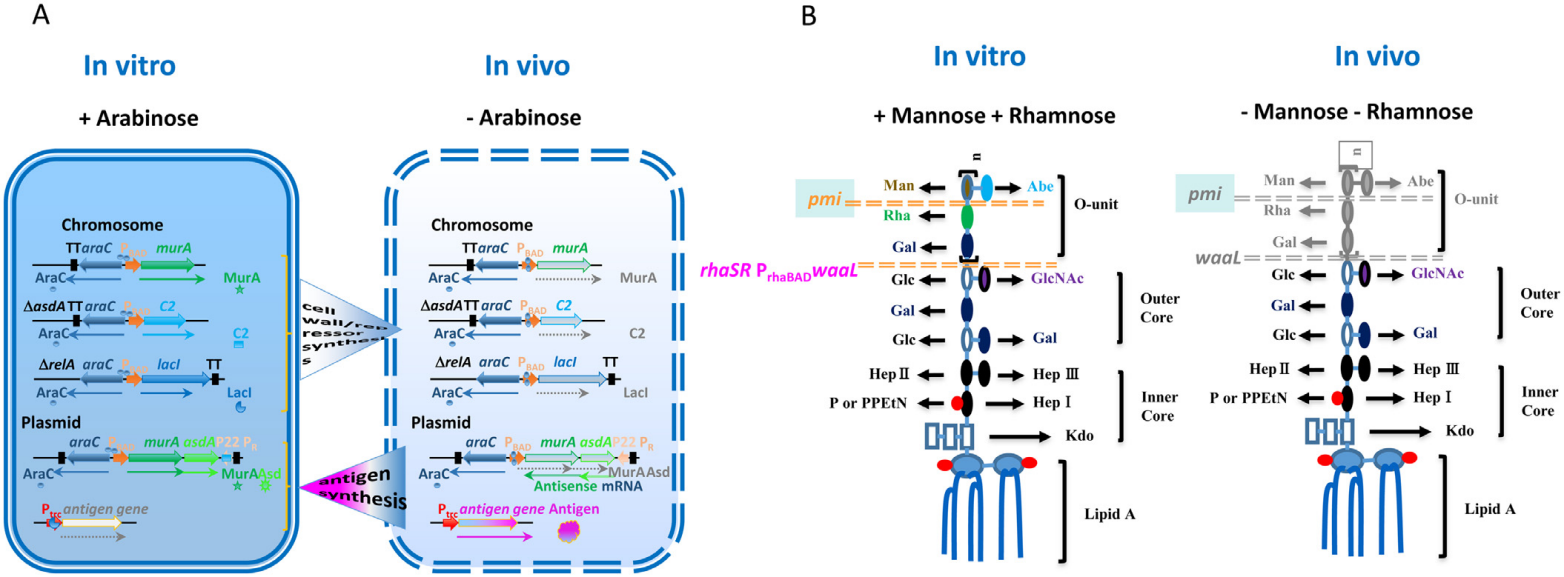
Principle features of a triple-sugar regulatable *Salmonella* vaccine. Arabinose- and rhamnose-regulated genes on chromosome and plasmid and mannose requirement will achieve regulated delayed lysis, regulated delayed attenuation, and regulated delayed antigen synthesis. (A) Arabinose-regulated genes. Arabinose regulates the expression of 4 genes, *murA, asdA, c2,* and *lacI.* Detailed information are in text. Gray color indicates the gene products that cease to be produced in vivo. (B) Mannose requirement and rhamnose-regulated genes. Mannose is required in strains with the Δ*pmi* mutation to synthesize O-antigen side chains in vitro. Rhamnose is required to synthesize WaaL to ligate O-antigen to core in vitro. Gray color indicates the gene products and structures that cease to be produced or formed in vivo. Abbreviations: Abe, abequose; Gal, galactose; Glc, glucose; GlcNAc, *N*-acetylglucosamine; Hep, heptose; kDa, kilodalton; Kdo, 3-deoxy-d-manno-octulosonic acid; Man, Mannose; P, phosphate; PPEtN, pyrophosphorylethanolamine.

The strain χ11802 is a 2-sugar, arabinose and mannose, regulatable vaccine vector strain that with a regulated lysis plasmid becomes a composite 2-sugar regulated vaccine vector (Figure 1). Arabinose regulates the expression of four genes, *murA, asdA, c2*, and *lacI*, each controlled by the P_araBAD_ promoter to achieve regulated delayed attenuation (Curtiss et al., 2009), regulated delayed lysis (Kong et al., 2008), and regulated delayed antigen synthesis (Wang et al., 2010; Figure 1A). Both MurA and Asd are used for regulated delayed lysis and attenuation. MurA (UDP-*N*-acetylglucosamine enolpyruvoyl transferase) is the first enzyme in the synthesis of muramic acid for the assembly of peptidoglycan. Its production is dependent on the presence of arabinose in the growth medium for χ11802 and ceases to be synthesized in vivo due to the absence of arabinose in internal animal tissues (Kong et al., 2008). MurA decreases as a consequence of cell division in vivo to ultimately lead to programmed cell lysis and death to enable biocontainment. The *murA* defect is complemented by an arabinose-regulated *murA* on a plasmid vector (Kong et al., 2008). Asd (aspartate semialdehyde dehydrogenase) is also involved in the biosynthesis of the bacterial cell wall. Deletion of *asdA* can be complemented by an arabinose-regulated *asdA* on a plasmid vector (Kong et al., 2008). To simplify the system, an arabinose-regulatable operon fusion of *murA* and *asdA* is placed on an expression plasmid to complement the deficiency caused by the chromosomal mutations (Figure 1A). The arabinose-dependent synthesis of the C2 repressor is to enable a regulated delayed expression of DNA sequences under the control of a promoter repressed by C2 (Kong et al., 2008). C2 can repress the P22 P_R_ promoter, which is on the expression plasmid with an opposite direction at the 3′ end of the *asdA* gene. When arabinose is absent, the P_R_ promoter will be derepressed to direct synthesis of antisense mRNAs of *asdA* and *murA* to block translation of any residual mRNA transcribed from these genes during programmed lysis. Strain χ11802 adopted a regulated delayed antigen synthesis system (Wang et al., 2010). This system, characterized by a deletion-insertion mutation Δ*relA*::*araC* P_araBAD_ *lacI*, enables repression of antigen production under the control of P_trc_ on an expression vector by arabinose regulated *lacI* expression in vitro with de-repression of antigen production in vivo as a consequence of vaccine strain cell division in the absence of arabinose (Wang et al., 2010). Strain χ11802 has the mutation Δ*relA198*::*araC* P_araBAD_ *lacI* TT to achieve the highest repression level in vitro and the slowest rate of derepression as a consequence of cell division *in vivo* (Wang et al., 2010). In strain χ12341, the mutation Δ*relA198*::*araC* P_araBAD_ *lacI* TT is replaced with Δ*relA197*::*araC* P_araBAD_ *lacI* TT (Wang et al., 2010), which produces moderate levels of LacI. This replacement enables an earlier derepression of antigen gene transcription for synthesis and delivery of antigens to the host immune system (Wang et al., 2010). The *pmi* gene encodes phosphomannose isomerase needed to interconvert fructose-6-P and mannose-6-P (Collins et al., 1991), which can be converted to GDP-Mannose for the synthesis of lipopolysaccharide (**LPS**) O-antigen side chains, to constitute another regulated delayed attenuation system (Curtiss et al., 2009) (Figure 1B). Free mannose (nonphosphorylated) in sufficient quantity is not available in animal tissues to support a level of LPS O-antigen synthesis for the display of a wild-type level of invasiveness and virulence (Collins et al., 1991). Strains with a Δ*pmi* mutation grown in media with mannose synthesize wild-type levels of O-antigen side chain at the time of immunization and exhibit nearly wild-type attributes for survival and colonization of lymphoid tissues. After eight to ten cell divisions in vivo they become avirulent due to the inability to synthesize the LPS O-antigen side chains (Curtiss et al., 2007). *S.* Typhimurium strains with the Δ*pmi* mutation are highly immunogenic, efficacious in enhancing induction of high antibody titers to cross-protective outer membrane proteins, and enhance the production of Outer Membrane Vesicles that can also deliver recombinant antigens to enhance induction of protective immunity (Muralinath et al., 2011). However, strains with the Δ*pmi* mutation do not completely expose the LPS core because there are still 2 sugars attached to the LPS core. In consideration of this potential problem, the mutation, Δ*pagL64*::TT *rhaRS* P_rhaBAD_ *waaL* (Figure 1B) was generated in strain χ12341*.* O-antigen ligase WaaL is necessary to ligate polysaccharide to the lipid A-LPS core moiety. Mutation of *waaL* results in an intact LPS core with no O-antigen or O-antigen sugars attached to it (Nagy et al., 2008). The entire *waaL* gene in the chromosomal O-antigen operon was deleted in a manner that did not alter the expression of adjacent genes in the operon. A rhamnose-regulated *waaL* (ΔP_rhaBAD_ *waaL*) was placed in the *pagL* gene since the *pagL* mutation does not impair *Salmonella* virulence (Kong et al., 2011a). Rhamnose regulation achieves better downregulation of O-antigen synthesis in vivo than does arabinose regulation because a relatively higher concentration of rhamnose is necessary to activate the P_rhaBAD_ promoter than needed for arabinose to activate the P_araBAD_ promoter (Brenneman et al., 2013). PIESV strains with rhamnose-regulated *waaL* will synthesis normal LPS in the presence of rhamnose in vitro but form rough LPS due to the absence of O-antigen ligase in vivo and expose the LPS core. The further truncated LPS will result in effective presentation of conserved outer membrane proteins and vectored antigens to the host immune system to enhance immunogenicity by increased phagocytosis (Nagy et al., 2008). Besides, loss of the LPS, a dominant surface antigen, will increase the immunogenic potential of vector antigens (Nagy et al., 2008). Thus, the PIESV strain χ12341 is a triple-sugar regulatable strain, which requires arabinose, mannose, and rhamnose to grow and display wild-type virulence. Strain χ12341 also has an additional Δ*sifA* mutation, which enables *Salmonella* to escape the endosome (termed the *Salmonella* containing vesicle, **SCV**) for lysis in the cytosol (Beuzón et al., 2000). Previous work demonstrated that a vaccine vector strain with the Δ*sifA* mutation conferred higher levels of protection when compared with a *sifA*^+^ strain against influenza virus and *Eimeria* challenges (Ashraf et al., 2011; Kong et al., 2020). These collective changes in χ12341 compared to χ11802 should enhance the attenuation, efficiency of lysis and biocontainment, and immunogenicity against clostridial antigens.

### Characterization of *Salmonella* Vaccine Strains

**Figure 2:**
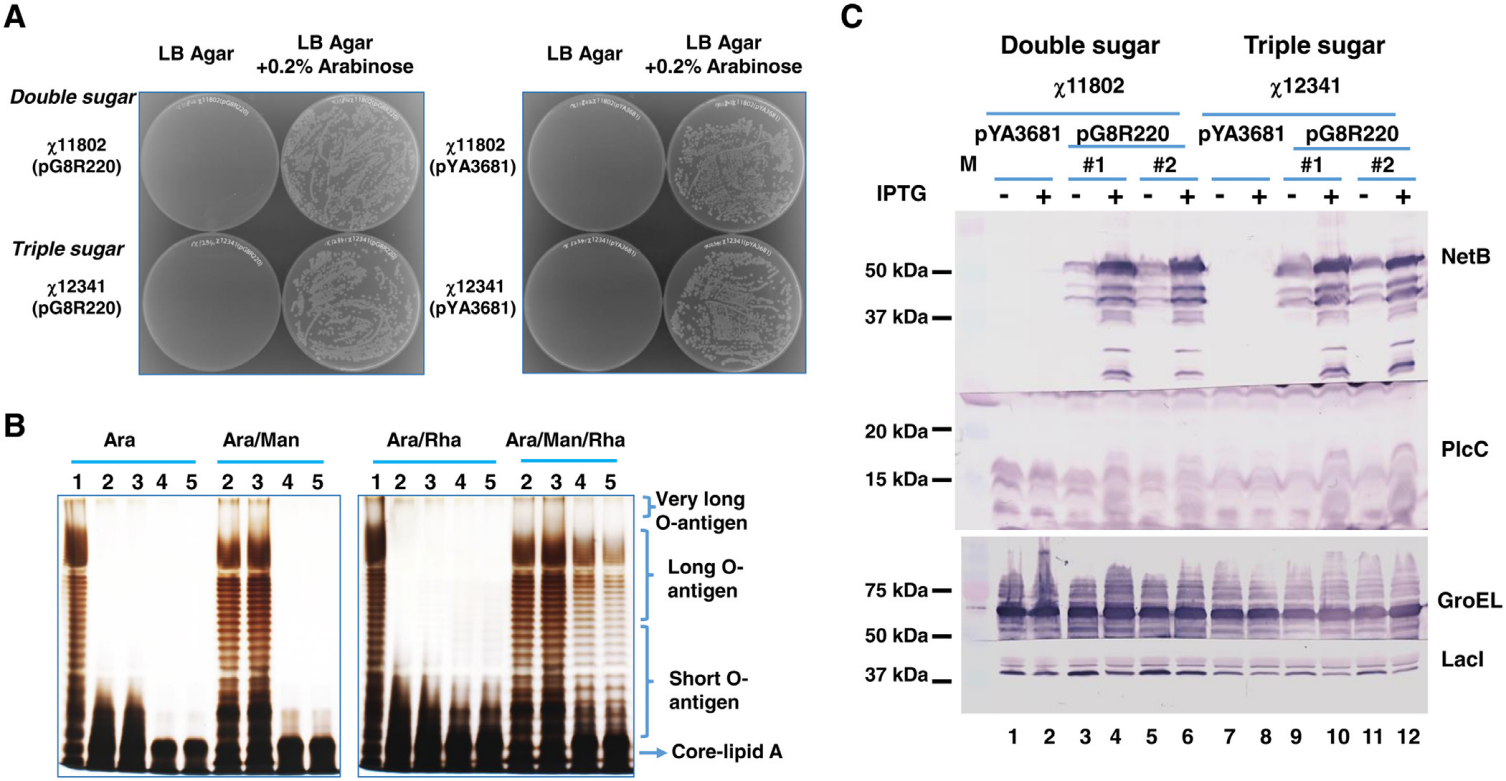
Phenotypic characterization of a double-sugar regulatable strain χ11802 and a triple-sugar regulatable strain χ12341. (A) The growth of strains χ11802 and χ12341 with vector pG8R220 or pYA3681 on LB agar plates with or without 0.2% arabinose. (B) LPS gels of strains χ11802(pG8R220) and χ12341(pG8R220) grown in Purple broth with indicated supplements. Lane 1, *Salmonella* wild-type strain χ3761; Lane 2, χ11802(pYA3681); Lane 3, χ11802(pG8R220); Lane 4, χ12341(pYA3681); Lane 5, χ12341(pG8R220). (C) Antigen production by vaccine strains as determined by western blots. Vaccine strains were grown in LB broth with necessary supplements as described in Materials and Methods. Antigen production was induced by the addition of 1 mM IPTG 4 h prior to harvest. Equal amounts of bacteria were collected for analysis. GroEL was used as a loading control. Membranes were probed with the indicated anti-sera. Predicted masses of antigens are: PlcC, 18 kDa; GST-NetB, 59 kDa; GroEL, 57 kDa; LacI, 39 kDa. #1 and #2, cultures from two colonies from each strain were analyzed.

Both double-sugar and triple-sugar regulated vaccine vector strains were transformed with plasmid pG8R220. The plasmid pG8R220, derived from pYA3681, is similar to plasmid pYA5112 (Jiang et al., 2015), which encodes an operon fusion for the synthesis of PlcC and GST-NetB as *C. perfringens* antigens. As expected, both *S.* Typhimurium strains χ11802 and χ12341 with control vector pYA3681 or pG8R220 displayed arabinose-dependent growth (Figure 2A). Strain χ11802 with either pYA3681 or pG8R220 requires both arabinose and mannose to synthesize complete O-antigen (Figure 2B, lanes 2 and 3), while χ12341 with either plasmid requires rhamnose in addition to these 2 sugars (Figure 2B, lanes 4 and 5). Strain χ12341 only synthesized the LPS core in the absence of rhamnose, and full O-antigen units similar to χ11802 when grown with mannose and rhamnose (Figure 2B, lanes 4 and 5). Production of PlcC and GST-NetB by the two strains were similar (Figure 2C). The amount of LacI produced by strain χ12341 was less than χ11802 due to replacing the Δ*relA198*::*araC* P_araBAD_ *lacI* TT by the Δ*relA197*::*araC* P_araBAD_ *lacI* TT deletion-insertion mutation (Figure 2C, lanes 7-12 vs. lanes 1-6). Corresponding to this result, the repression of the NetB protein produced in the uninduced state of strain χ12341(pG8R220) was slightly weaker than χ11802(pG8R220) (Figure 2C, lanes 9, 11 vs. lanes 3, 5). However, this difference did not affect the growth of χ12341(pG8R220) (data not shown). All vaccine strains showed similar growth characteristics in LB broth with necessary supplements. After growth for 50 generations under permissive conditions with arabinose and DAP (Juárez-Rodríguez et al., 2012), all the strains kept their plasmids and could synthesize antigens as expected (data not shown).

### Evaluation of Protection in Broiler Chickens Against *C. Perfringens* Challenge

#### Trial 1

The triple-sugar regulated strain induced better protection than the double-sugar regulated strain (Table 2).

**Table 2:** Protective immunity induced by a double-sugar regulated strain χ11802 and a triple-sugar regulated strain χ12341.

	NE	% NE	Feed conversion		Weight gain (kg)		Feed conversion		Weight gain (kg)	
Treatments	Lesions	Mortality	D 0–21	D 14–21	D 0–21	D 14–21	D 0–28	D 14–28	D 0–28	D 14–28
1. NV/NCh	0.0^d^	0.0^a^	2.05^b^	1.63^c^	0.29^a^	0.16^a^	1.96^b^	1.71^b^	0.66^a^	0.54^a^
2. NV	0.9^a^	6.3^a^	2.59^a^	2.05^ab^	0.23^b^	0.13^b^	2.24^a^	1.88^ab^	0.51^b^	0.41^b^
3. χ11802(pG8R220)	0.8^a^	6.3^a^	2.34^ab^	2.09^a^	0.27^ab^	0.14^ab^	2.10^ab^	1.89^a^	0.63^ab^	0.50^ab^
4. χ12341(pG8R220)	0.3^cd^	0.0^a^	2.16^b^	1.83^bc^	0.29^a^	0.15^a^	1.94^b^	1.70^b^	0.71^a^	0.57^a^
5. Vector Control	0.6^ab^	4.7^a^	2.32^ab^	2.04^ab^	0.28^ab^	0.15^a^	2.06^ab^	1.85^ab^	0.65^a^	0.53^a^
6. BMD	0.5^bc^	1.6^a^	2.24^b^	1.89^ab^	0.27^ab^	0.15^a^	1.98^b^	1.74^ab^	0.64^a^	0.53^a^

abcd Group data with the same letters were not significantly different. All groups were challenged with *C. perfringens* CP6 except Group 1 NV/NCh. NV were nonmedicated group.

The first trial was to compare the protective immunity induced by strain χ11802 (arabinose- and mannose-regulatable phenotypes) and χ12341 (arabinose-, mannose- and rhamnose-regulatable phenotypes) containing plasmid pG8R220 in an *Eimeria-C. perfringens* challenge model after a single oral dose immunization. The test was carried out in day-of-hatch chickens, which is different from previous tests in 3-day-old chickens with a two-dose oral immunization regime (Jiang et al., 2015; Wilde et al., 2019). The experiment also included a BMD positive control and a NV/NCh negative control, which were not included in previous evaluations (Jiang et al., 2015; Wilde et al., 2019). The *Eimeria* stress model was used since *Eimeria* co-infection causing coccidiosis has a high impact on the occurrence and severity of NE (Prescott et al., 2016). The identities of vaccine strains used in these trials were blinded to the SPRG staff. None of the vaccinated chickens showed clinical signs before challenge. After challenge, the lesion scores in the χ11802**(**pG8R220) immunized group (Treatment 3) were similar to NV (Treatment 2) and Vector Control groups (Treatment 5), which were significantly higher than the lesion scores in the χ12341**(**pG8R220) immunized group (Treatment 4), the NV/NCh (Treatment 1) and BMD (Treatment 6) groups. The lesion scores in the χ12341**(**pG8R220) immunized group were similar to those in the BMD and NV/NCh groups. After challenge, the mortality in chickens immunized with χ11802**(**pG8R220) was the same as for the NV group. Although there was no significant difference among challenge groups, the chickens immunized with χ12341**(**pG8R220) showed no mortality, which was the same as observed for the NV/NCh group. Other groups including the BMD group showed low mortality.

Reduced weight gain and increased feed conversion ratio are indications of subclinical *C. perfringens* infections. The χ12341(pG8R220) group had similar feed conversion and weight gain levels compared to the χ11802(pG8R220), NV/NCh, Vector Control, and BMD groups between D 0–21 and D 0–28. After challenged with *C. perfringens,* the χ12341(pG8R220) group still had similar feed conversion compared to the NV/NCh and BMD groups between D14-21 and D14-28, but better than the χ11802(pG8R220) group, which had similar feed conversion values compared to the NV and Vector Control groups. Chickens immunized with χ12341(pG8R220) showed the highest weight gain between D 0–28 and D 14–28. The protective effect conferred by χ12341**(**pG8R220) was superior (lesion score) or similar (mortality, weight gain and feed conversion) to the industry standard antibiotic treatment group (BMD group). Although there were no differences in overall (D0-28) feed conversion and weight gain between the χ12341(pG8R220) and Vector Control groups, the χ12341(pG8R220) group was different from the NV group, while the Vector Control group was the same, suggesting that the χ12341 group provides more advantage than observed in the Vector Control group. In summary, lesion score showed there were significant differences between the χ12341(pG8R220) immunized group and other challenge groups. Weight gain and feed conversion ratio data showed that there was no significant differences between χ12341(pG8R220), NV/NCh or BMD groups. These results indicated that the triple-sugar regulated strain χ12341 was superior to the double-sugar regulated strain χ11802 in inducing protection against *C. perfringens* challenge after a single oral vaccination.

#### Trial 2: Dose Effect of PIESV χ12341(pG8R220)

After showing that the triple-sugar regulated strain was superior to the double-sugar regulated strain, a new trial was conducted to assess the effect of the oral immunizing dose of χ12341(pG8R220; Table 3). The experiment was performed with low (5 × 10^7^ CFU, LD Gavage), intermediate (1 × 10^8^ CFU, MD gavage), and high (5 × 10^8^ CFU, HD Gavage) doses of χ12341(pG8R220; Table 3). Trial 2 resulted in 15.6% mortality in the NV group. It might be because the *Eimeria* oocyst population used in this trial was more potent than that used in Trial 1 in initiating intestinal damage to augment seriousness of disease after *C. perfringens* challenge. With this high mortality, the lesion scores for the different doses of χ12341(pG8R220) were similar to those for the Vector control, BMD, and the NV groups. Although the *C. perfringens* challenge resulted in higher mortality in the NV group than that in Trial 1 (15.6 vs. 6.3%), all *Salmonella* immunized groups and the BMD group displayed significantly lower mortality than that in the NV group. HD and MD Gavage groups were similar to the BMD group. Weight gain and feed conversion data demonstrated that there were no differences between the HD and MD Gavage groups and the BMD group, with both HD and MD Gavage groups superior to the NV group during D 14–21 and D 14–28 (except for weight gain in the MD Gavage group during D 14–28). After challenge, the weight gain of the HD Gavage group was higher than that of the NV and Vector Control groups during D 14–28. These mortality data indicated that the *Salmonella*-induced protection against CP6 challenge was dose-related. It was noticed that the weight gains of the HD Gavage group were the same as in the Vector Control group between D 0–28 and D14–28. However, there were weight gain differences between the HD Gavage and NV groups that were the same between the Vector Control and NV groups. The results implied that the HD Gavage group displays an advantage over the Vector Control group.

**Table 3:** Dose-effect of PIESV χ12341(pG8R220) against *C. perfringens* CP6 challenge.

	NE	% NE	Feed conversion		Weight gain (kg)		Feed conversion		Weight gain (kg)	
Treatments	Lesions	Mortality	D 0–21	D 14–21	D 0–21	D 14–21	D 0–28	D 14–28	D 0–28	D 14–28
1. NV/NCh	0.1^b^	0.0^c^	1.71^c^	1.84^d^	0.51^a^	0.22^a^	1.81^b^	1.92^c^	0.71^a^	0.42^a^
2. NV	0.4^ab^	15.6^a^	2.33^a^	3.19^a^	0.35^c^	0.12e	2.21^a^	2.52^a^	0.50^c^	0.27^d^
3. HD gavage	0.4^a^	1.6^bc^	2.26^ab^	2.20^cd^	0.40^b^	0.18^b^	2.16^a^	2.07^bc^	0.60^b^	0.38^ab^
4. MD gavage	0.5^a^	1.6^bc^	2.24^ab^	2.44^bc^	0.39^bc^	0.15^cd^	2.12^a^	2.17^bc^	0.57^bc^	0.33bcd
5. LD gavage	0.5^a^	6.3^b^	2.39^a^	2.42^bc^	0.38^bc^	0.16bcd	2.23^a^	2.13^bc^	0.57^bc^	0.36abc
6. Vector control	0.5^a^	4.7^bc^	2.31^a^	2.75^b^	0.38^bc^	0.14^d^^e^	2.17^a^	2.29^ab^	0.54^bc^	0.30^cd^
7. BMD	0.5^a^	1.6^bc^	2.04^b^	2.14^cd^	0.41^b^	0.17^bc^	2.04^a^	2.10^bc^	0.59^bc^	0.35abc

abcd Groups with the same letters were not different. All groups were challenged with *C. perfringens* CP6 except Group 1 NV/NCh. NV was nonmedicated group, HD, MD, and LD were high, intermediate and low doses of χ12341(pG8R220).

#### Trial 3

Effect of doses and immunization routes of χ12341(pG8R220) (Table 4).

**Table 4:** Effects of the route of immunization of χ12341(pG8R220) against *C. perfringens* CP4 challenge.

	NE	% NE	Feed conversion		Weight gain (kg)		Feed conversion		Weight gain (kg)	
Treatments	Lesions	Mortality	D 0–21	D 14–21	D 0–21	D 14–21	D 0–8	D 14–28	D 0–28	D 14–28
1. NV/NCh	0.0^c^	0.0^c^	1.68^d^	1.88^d^	0.52^a^	0.24^a^	1.77^c^	1.94^d^	0.84^a^	0.55^a^
2. NV	0.9^a^	40.6^a^	1.98^a^	2.73^a^	0.45^bc^	0.16^d^	2.21^a^	3.27^a^	0.64^c^	0.35^c^
3. HD gavage	0.7^ab^	20.3^b^	1.96^ab^	2.54^ab^	0.44^c^	0.17^cd^	1.98^bc^	2.36^bc^	0.66^bc^	0.40^bc^
4. MD gavage	0.4^bc^	12.5^bc^	1.81bcd	2.21^c^	0.47^bc^	0.19^bc^	1.84^bc^	2.12bcd	0.77^ab^	0.50^ab^
5. HD spray	0.9^a^	20.3^b^	1.91^ab^^c^	2.49^b^	0.45^bc^	0.17bcd	1.86^bc^	2.19bcd	0.77^ab^	0.48^ab^
6. HD drinking water	0.8^ab^	20.3^b^	1.90abc	2.57^ab^	0.47^bc^	0.17^cd^	1.98^b^	2.51^b^	0.79^a^	0.49^ab^
7. BMD	0.4^bc^	17.2^b^	1.76^cd^	2.11^c^	0.49^ab^	0.20^b^	1.81^bc^	2.07^cd^	0.83^a^	0.54^a^

abcd Groups with the same letters were not different. All groups were challenged with *C. perfringens* CP4 except Treatment 1 NV/NCh. NV was nonmedicated group, HD and MD were high and intermediate doses of χ12341(pG8R220).

To further evaluate the vaccine strain χ12341(pG8R220), chicks were immunized with either a high dose (5 × 10^8^ CFU) or intermediate dose (1 × 10^8^ CFU) of the χ12341(pG8R220) strain by oral gavage, by spray or in drinking water. The Vector Control group was dropped because of the limited facility capacity and its disadvantage as observed in Trials 1 and 2. Broiler vaccines are generally delivered via coarse spray in the hatchery or via drinking water in broiler houses (Aehle and Curtiss, 2017) in addition to in ovo immunization. A virulent *C. perfringens* strain CP4, which leads to 40% mortality in nonvaccinated (NV) broilers challenged with this strain, was used as the challenge strain to evaluate the broad effectiveness of the vaccine (Table 4). This trial showed very high mortality under these challenge conditions in the NV group. All routes of immunization were superior to the NV group relative to mortality, with a 75 to 50% reduction, and the same to BMD control group. The MD Gavage group showed a significantly lower lesion score than the NV group, which was similar to the BMD and NV/NCh groups (Table 4). The spray and drinking water immunization groups are less protective than the MD Gavage group in reducing lesion scores but similar to the HD Gavage group in reducing mortality.

The MD Gavage group displayed similar feed conversion and weight gain to the NV/NCh and BMD groups during D 0–28 and D 14–28, indicating that the MD Gavage dose did not adversely affect the growth of the chickens. The HD Gavage group showed higher feed conversion and lower weight gain than BMD groups during D 0–21, D 14–21, indicating that the HD Gavage dose might slightly affect growth before D 21. Although the HD Gavage group had similar feed conversion to the BMD group during D 0–28 and D14–28, it had less weight gain than the BMD group during these time frames. Both the HD Spray and HD Drinking Water groups had similar feed conversion and weight gain to the BMD group during D 0–21 and D 0–28. Both groups had similar weight gains to the BMD group during D 0–21, D 0–28, and D 14–28, indicating that these 2 immunization routes did not affect the growth of chickens. These results indicated that MD Gavage is an optimal immunization dose and route for χ12341(pG8R220) against 1 different challenge strains, CP4 and CP6. This is most likely due to the fact that every chick vaccinated by Gavage gets the same dose of vaccine whereas vaccination by spray or in drinking water results in a larger variation in doses actually exposed to or taken up by chicks in these populations.

## DISCUSSION

The ultimate goal of this project was to design an attenuated *Salmonella* strain that could be used as a highly efficient vector to deliver multiple antigens to induce protective immunity against *C. perfringens* infection to curtail the induction of NE. Previous reports demonstrated that either PlcC or NetB alone confers protection (Lee et al., 2011). We are the first to deliver these 2 antigens simultaneously to induce protection in the consideration that Type G *C. perfringens* has both α- and NetB toxins (Jiang et al., 2015). Our previous data proved that the operon fusion of nontoxic antigens PlcC and GST-NetB delivered by *Salmonella* induce protection (Jiang et al., 2015; Wilde et al., 2019). The effectiveness of the fusion of PlcC and NetB has also been demonstrate by other researchers (Hunter et al., 2019; Katalani et al., 2020). *Salmonella* delivering fructose-1,6-bisphosphate aldolase, has also been proved to induce protection against *C. perfringens* challenge (Wilde et al., 2019). The above results laid the foundation for further improvement of the vaccine. Beside different immunization regimes, such as maternal and in ovo immunization, we speculated that improvement of the *Salmonella* vector strain, based on ongoing work in our lab, would provide a better mean to improve vaccine efficacy. This hypothesis was reasonable since it was well-established that the operon fusion of PlcC and Gst-NetB were effective (Jiang et al., 2015; Wilde et al., 2019). Once an improved *Salmonella* vector was developed, it could be used to deliver more antigens by course spray/drinking water vaccination or be tested as a maternal vaccine or for in ovo vaccination.

The broiler life span is only 6 to 7 wk, posing a challenge for vaccines to induce protective immunity after hatch. Immunization of day-of-hatch chickens is envisaged as a practical way for field application. In this consideration, the antigen should be produced to induce immune response once the *Salmonella* vaccine reaches the immunocompetent tissues in the immunized broiler after vaccination. Live *Salmonella* vectored vaccines that colonize internal effector lymphoid tissues serve as factories to multiply, disseminate, produce, and deliver antigens until lysis exceeds multiplication and dissemination (Curtiss et al., 2010). Thus, protective antigens are delivered for a week or more to stimulate immune system. We have achieved this objective by a better selection of some mutations in χ12341. First, the replacement of Δ*relA198*::*araC* P_araBAD_ *lacI* TT with Δ*relA197*::*araC* P_araBAD_ *lacI* TT mutation enables the derepression of antigen gene transcription and synthesis of antigens 3.3 generations earlier (Wang et al., 2010). Although direct verification of the derepression time is impossible since both strains require arabinose to survive, the repressed levels of PlcC and NetB in χ12341 were less than in χ11802, corresponding to reduced levels of LacI in χ12341 (Figure 2), indicating that this could be the expected scenario in vivo. The second mutation is the Δ*sifA* mutation based on previous evidence demonstrated in influenza challenge (Ashraf et al., 2011; Kong et al., 2012) and in other studies on inducing protective immunity against *Eimeria* challenge (Kong et al., 2020). The release of antigen due to programmed lysis within the SCV would likely stimulate an MHC II antigen presentation, dendritic cell migration, and adaptive immune responses (Mitchell et al., 2004; Halici et al., 2008). The Δ*sifA* mutation should also increase the interaction between released antigens and MHC I to enhance the induction of CD8-mediated immune responses. The third modification in χ12341 compared to χ11802 consists of 2 mutations Δ*pagL64*::TT *rhaRS* P_rhaBAD_ and Δ*waaL* enabling synthesizing complete LPS O-antigen in vitro in addition to the Δ*pmi-2426* mutation that is also present in χ11802*.* Structurally rough mutants defective in the synthesis of the LPS core or O-antigen are not considered appropriate as live attenuated vaccine candidates (Kong et al., 2011b). However, the regulated synthesis of LPS in vaccine strains could overcome these shortcomings (Curtiss et al., 2007). The mutations, Δ*pagL64*::TT *rhaRS* P_rhaBAD_ and Δ*waaL*, enable χ12341 to display a rough phenotype with an intact LPS core with no O-antigen attached to it when grown without rhamnose (Figure 2), which is the situation the vaccine strain will meet in vivo. The completely O-antigen deprived bacteria will better expose the LPS core to the host immune system and to increase phagocytosis by host immune cells. Abolished production of dominant and variable LPS should also increase the immunogenic potential of delivered antigens (Nagy et al., 2008). The introduction of these mutations provides a complementary pathway for the Δ*pmi-2426* mutation to regulate O-antigen production*.*

Hosts respond to pathogens in multiple ways by responses of the immune, nervous and endocrine systems. The immune parameters are mainly adopted since the means to monitor other responses not associated with the immune system have not been studied or described. We previously showed that IgY against PlcC decreased the growth of *C. perfringens* but only afforded partial protection (Zekarias et al., 2008) and mucosal immune responses were important for the protection against necrotic enteritidis, but did not directly correlate to the protection observed (Jiang et al., 2015). Others also reported the uncertainties about the role of antibody associated-protection (Kulkarni et al., 2007; Jiang et al., 2009; Kulkarni et al., 2010; Jang et al., 2012; Mot et al., 2014). The mode of action is not going to be a systemic immune response and primarily against the toxins that allow the *C. perfringens* to proliferate, therefore protection is the best measure of efficacy by far. In practical usage, mortality, feed conversion and weight gain are important economic considerations for the boiler industry. We used all 3 indexes to evaluate the practical usage of our vaccine in addition to lesion score. The operon fusion of the PlcC-GST-NetB reduced the severity of necrotic lesion in a high-protein feed model (Jiang et al., 2009). The χ11802 carrying a similar construction did not induce a similar protective level in the *Eimeria-Clostridium* challenge model. NE causes mortality in peracute and acute forms in poultry. Ideally, the NE challenge model should be reproducible and resemble the situation described in the field. The challenge model used here mimic the farm condition that *Eimeria* is one of the predisposing factors for *C. perfringens* induced NE. Coccidia combined with *C. perfringens* produces more severe NE than that with a high-protein diet because coccidia cause damage to the epithelium to facilitate the development of NE. It is a more severe challenge model than the high-protein feed model. However, the same construction delivered by the improved strain χ12341 induced a high level of protection, proving the effectiveness of χ12341. In trials 2 and 3 with this model, we observed variation of NE scores in the NV group compared to the BMD group, but consistently higher mortality than in the BMD group. However, the mortality was not directly proportional to lesion scores of surviving birds. This is partly because the lesions in dead birds were not included in the lesion score data, but only in mortality to avoid the repeat calculation. Thus, the *Eimeria-Clostridium* challenge model seems to mimic the acute infection of *C. perfringens* better, but not for subclinical infection. With this severe disease model, we observed similar weight gain and feed conversion efficiency between vaccine and NV groups in most of our tests. These data support the potential of our vaccine to be used to prevent acute infections. The vaccine construct described here has been evaluated in multiple field trials with lots of 50,000 broilers and routinely showed a significant reduction in the overall low level of mortality observed as likely due to subclinical infections with *C. perfringens.*

Using this *Eimeria-Clostridium* challenge model, strain χ12341 was better than strain χ11802 to reduce lesion scores without compromising feed conversion efficiency and weight gain (Table 2). This strain also displayed good biocontainment, with no bacterial shedding after 6 d. Though strain χ12341 is effective in the multiple tests with different doses and immunization routes (Table 2, Table 3, Table 4), further optimization to decrease the rate of O-antigen loss in vivo might be beneficial. We also observed that the MD Gavage group conferred similar protection as the HD Gavage group. Intuitively, the HD Gavage should be better than the MD gavage. Since this is a live vectored vaccine, with the increase of the dose, both *Salmonella* and *C. perfringens* antigens delivered increase. However, there will be more antigens to *Salmonella* increased than to *C. perfringens.* This could lead to diverting the immune responses to respond more to *Salmonella* antigens than to *C. perfringens* antigens, which could be tested in the future. The portion of the responses against *C. perfringens* antigens in the HD Gavage should be less than those in the MD Gavage. In this consideration, use of the MD Gavage is enough to induce immune responses to focus on the *C. perfringens* antigens. In another way, both high and low amounts of antigen might induce B-cell unresponsiveness in mice and lead to exhaustive induction of immune responses (Bachmann and Zinkernagel, 1997). A medium dose might be suitable.

We also observed that the *Salmonella* harboring empty vector vaccinated group tended to have a lower lesion score than the NV group (Table 2). This is not unique in this trial. Our previous work with influenza (Ashraf et al., 2011; Kong et al., 2012), *Mycobacterium tuberculosis* (Juárez-Rodríguez et al., 2012), *Yersinia* spp. (Branger et al., 2009), *Streptococcus pneumoniae* (Nayak et al., 1998) and *Eimeria* spp. (Kong et al., 2020) also showed PIESVs with an empty vector giving greater levels of protection compared to administering buffer saline. *Salmonella* has multiple ways to activate innate immune responses through pathogen associated molecular patterns (**PAMPs**) and damage associated molecular patterns (**DAMPs**), so that these strains can act as potent adjuvants. The activation of innate immunity is critical to elicit adaptive immune responses. Although PIESVs are not designed to stimulate the innate immune system, the PIESV are programmed to undergo regulated lysis in various cell compartments to maximize delivery of DAMPs and PAMPs, such as flagellin, CpG, peptidoglycan components, DNA, RNA, ATP, lipoprotein, to activate the innate immune system through interaction with pattern recognition receptors to result in optimal recruitment of innate immune cells (Kong et al., 2008; Higgins and Mills, 2010; Kawasaki and Kawai, 2014). The activated innate immune responses enable some hosts to survive the challenge infection for sufficient time to stimulate an acquired immune response and develop protective immunity. In this case, antigen delivery by regulated delayed lysis gives superior immune responses compared to delivery without program lysis in different studies (Kong et al., 2008; Ashraf et al., 2011; Juárez-Rodríguez et al., 2012; Jiang et al., 2015).

In summary, an improvement over the double-sugar regulated vaccine strain χ11802, an early generation backbone with published evidence of induced protection, was developed by using triple-sugar regulation of vaccine attributes. The triple-sugar regulated *Salmonella*-*C. perfringens* vaccine is safe and effective. In multiple studies using a single oral vaccination of the day-of-hatch broiler chickens using χ12341(pG8R220), the triple sugar-regulated strain either reduced mortality or reduced intestinal lesions in an *Eimeria*-*C. perfringens* challenge model*.* The protective effect was dose-dependent and could be achieved through multiple immunization routes. The improved body weights and feed conversion ratios were similar to those in chickens provided with the standard antibiotic treatment used to control NE and at levels similar to those in the NV/NCh group. Regardless of the variables in *C. perfringens* challenge strain, vaccine route and/or vaccine dose, the χ12341(pG8R220) candidate vaccine demonstrates consistent protection from the effects of *C. perfringens* challenge at a level equal to or superior than BMD treatment. This vaccine will pave the way to develop a low-cost solution to *C. perfringens* caused NE.
